# A phase 2 randomized, double-blind, placebo-controlled study of tremelimumab for second and third line treatment in patients with unresectable pleural or peritoneal mesothelioma

**DOI:** 10.1186/2051-1426-1-S1-P132

**Published:** 2013-11-07

**Authors:** Lee Krug, Alessandra di Pietro, Rajesh Narwal, Paul Robbins, Dongyue Fu, Aiman Shalabi, Ramy Ibrahim, Luana Calabro, Hedy Kindler

**Affiliations:** 1 Memorial Sloan-Kettering Cancer Center, New York City, NY, USA; 2 MedImmune, LLC, Gaithersburg, MD, USA; 3 University Hospital of Siena, Siena, Italy; 4 The University of Chicago, Chicago, IL, USA

## Introduction

Malignant mesothelioma (MM) is an uncommon cancer, caused principally by asbestos exposure. No treatments after first-line platinum-pemetrexed [1] have shown survival benefit [2], thus novel approaches are needed. Asbestos exposure induces immunosuppression and immune dysfunction in the mesothelium environment, mainly by hyperactivation of regulatory T lymphocytes and over-production of cytokines that inhibit cytotoxic T lymphocytes and natural killer cells [3]. Cytotoxic T lymphocyte-associated antigen 4 (CTLA-4, CD152) modulates and eventually switches off T cell activation. Tremelimumab (treme) binds to the CTLA-4 antigen, preventing its negative regulatory signal to cytotoxic T cells. Results from a single arm phase 2 study of treme in 29 patients with MM who progressed on a platinum-based regimen showed promising 1- and 2- year survival rates and a safety profile consistent with previous treme studies [4]. This study has been expanded to enroll 29 patients currently treated with an optimized dosing schedule.

## Study design

This is a phase 2, randomized, double-blind, placebo-controlled study. Patients with unresectable pleural or peritoneal MM who progressed following 1or 2 prior treatments, including a first-line platinum-pemetrexed regimen, will be randomized in a 2:1 ratio to receive either treme or placebo. Randomization will be stratified by EORTC status (low- vs high-risk), line of therapy (second vs third), and anatomical site (pleural vs peritoneal). Enrollment will include 180 subjects at approximately 150 centers in multiple countries. Recruiting began in May 2013.

## Endpoints

Primary: overall survival. Secondary: durable disease-control rate (DCR); progression-free survival (PFS); patient-reported outcomes (pain, disease-related symptoms, and time to deterioration of disease-related symptoms); duration of response and overall response rate (ORR); treme safety profile, immunogenicity, and pharmacokinetics. Exploratory: DCR, PFS, duration of response and ORR based on immune related response criteria, health-related quality of life, disease-related symptoms, pain, and health status in patients with durable clinical activity. The association of biomarkers with treme and clinical outcomes will also be explored.
